# Thermal-responsive genetic and epigenetic regulation of *DAM* cluster controlling dormancy and chilling requirement in peach floral buds

**DOI:** 10.1038/s41438-020-0336-y

**Published:** 2020-08-01

**Authors:** Hong Zhu, Pao-Yang Chen, Silin Zhong, Chris Dardick, Ann Callahan, Yong-Qiang An, Steve van Knocker, Yingzhen Yang, Gan-Yuan Zhong, Albert Abbott, Zongrang Liu

**Affiliations:** 1 USDA-ARS, Appalachian Fruit Research Station, Kearneysville, WV 25430 USA; 2 Key Laboratory of South China Agricultural Plant Molecular Analysis and Genetic Improvement, South China Botanical Garden, Chinese Academy of Sciences, Guangzhou, 510650 China; 3 Institute of Plant and Microbial Biology, Academia Sinica, Taipei, 11529 Taiwan; 4 The State Key Laboratory of Agrobiotechnology, School of Life Science, The Chinese University of Hong Kong, Hong Kong, Hong Kong; 5 USDA-ARS, Plant Genetics Research Unit, Donald Danforth Plant Science Center, St Louis, MO 63132 USA; 6 Department of Horticulture, Michigan State University, East Lansing, MI 48834 USA; 7 USDA-ARS, Grape Genetic Research Unit, Geneva, NY 14456 USA; 8 Forest Health Research and Education Center, University of Kentucky, Lexington, KY 40546 USA

**Keywords:** Plant molecular biology, Epigenetics

## Abstract

The *Dormancy-associated MADS-box* (*DAM*) gene cluster in peach serves as a key regulatory hub on which the seasonal temperatures act and orchestrate dormancy onset and exit, chilling response and floral bud developmental pace. Yet, how different temperature regimes interact with and regulate the six linked *DAM* genes remains unclear. Here, we demonstrate that chilling downregulates *DAM1 and DAM3–6* in dormant floral buds with distinct patterns and identify *DAM4* as the most abundantly expressed one. We reveal multiple epigenetic events, with tri-methyl histone H3 lysine 27 (H3K27me3) induced by chilling specifically in *DAM1* and *DAM5*, a 21-nt sRNA in *DAM3* and a ncRNA induced in *DAM*4. Such induction is inversely correlated with downregulation of their cognate *DAM*s. We also show that the six *DAM*s were hypermethylated, associating with the production of 24-nt sRNAs. Hence, the chilling-responsive dynamic of the different epigenetic elements and their interactions likely define distinct expression abundance and downregulation pattern of each *DAM*. We further show that the expression of the five *DAM*s remains steadily unchanged or continuously downregulated at the ensuing warm temperature after chilling, and this state of regulation correlates with robust increase of sRNA expression, H3K27me3 and CHH methylation, which is particularly pronounced in *DAM4*. Such robust increase of repressive epigenetic marks may irreversibly reinforce the chilling-imposed repression of *DAM*s to ensure flower-developmental programming free from any residual *DAM* inhibition. Taken together, we reveal novel information about genetic and epigenetic regulation of the *DAM* cluster in peach, which will be of fundamental significance in understanding of the regulatory mechanisms underlying chilling requirement and dormancy release, and of practical application for improvement of plasticity of flower time and bud break in fruit trees to adapt changing climates.

## Introduction

Dormancy is an adaptation that enables perennial plants to survive unfavorable seasonal stresses. In the temperate zone where winter freezing is a major threat, plants enter the dormant state in late fall before winter to avoid freezing injury^1,2^. Seasonal environmental cues primarily dictate dormancy onset and development and release, which has been intensively studied in temperate perennials that undergo winter dormancy^1,3,4^. For example, a shortening photoperiod or declining temperature or both in the fall induce dormancy in peach (*P. persica* L. Batsch), while declining temperature serves as the only factor for apple (*M. domestica*) and pear *(P. communis*). In all cases, environmental cues cause the apical shoot meristem to cease growth and form a bud to enter the ecodormant state^1,5^. In contrast, lateral vegetative buds formed as result of apical dominant growth suppression, are in a state of paradormancy^6^. Both ecodormancy and paradormancy are temporary, reversible, and serve as initial stages for the transition into the deep dormant state called endodormancy in late fall^5^. The floral buds that initiate and form in the summer, similarly enter endodormancy near the end of fall^7^. Endodormancy is a physiological state that is not readily broken or released by short favorable environmental conditions unless exposed to chilling temperatures (>0–7.5 °C)^1^. This chilling requirement is obligatory, but varies considerably among plants, which is primarily determined by origin and genotype^8^. Chilling requirement also varies among different buds within the same trees or floral organs within the same flower as reflected by the longer chilling period required by the dormant floral buds compared to apical leaf buds^7^ and the female floral organ compared to the male organ^9^. Hence, the biological nature and developmental trajectory of meristems also contribute to chilling requirement.

Chilling is, in fact, essential for floral development. In contrast to dormant vegetative buds that are believed to primarily arrest at G1 phase of the cell cycle and remain quiescent during the chilling period^10^, the dormant peach floral buds undergo morphological changes^11^, with evident formation of distinct archesporial cells and epidermis, microsporangium walls and tapetum in the anthers^12,13^, and visible ovules in the carpel/gynoecium^14–16^. Yet, these floral morphological changes rarely occur in the fully dormant floral buds maintained at ambient or warm temperatures^11^. The morphological response is, in fact, chilling stage-dependent and major development events such as ovule formation in carpel only occurs near the end of the chilling period^12–16^, and insufficient chilling leads to the arrest of carpel development before or at the stage of ovule formation^9^. Evidently, chilling couples the dormancy release with floral developmental programming and only critical stages (e.g. ovule formation) driven through by chilling renders the floral buds capably released from dormancy or competent to grow in spring.

Warm temperature immediately following chilling plays an important role in the coordination of floral bud development and break. Even after chilling requirement is fulfilled, the floral buds still do not immediately progress to flowering unless exposed to a period of warm temperatures, a phenomenon termed “heat sum” requirement, which has been documented in numerous temperate deciduous fruit trees^17–19^. Like the chilling requirement, the heat requirement is highly heritable and often ecotypically adapted^20^. However, temperatures at or above 25 °C often impair floral organ development^21,22^. Chilling and warm requirements are interrelated and interact such that longer chilling periods lead to a shorter warm period requirement, suggesting common genes or pathways are targeted by both temperature regimes. In contrast, the warm requirement is directly related to bud break and flowering time and cannot be completely substituted by chilling^10,18^.

Dormancy onset and exit, and chilling and warm requirement appear to share a similar genetic regulatory basis, which is supported by the pioneering study on the characterization of a peach *evergrowing* (*EVG*) mutant that loses dormancy in both apical shoot meristems and floral buds^23^. Genome analysis revealed six tandemly duplicated highly conserved *dormancy-associated MADS-BOX genes (DAM1-6)* located in the *Wt EVG* locus, and identified a large deletion removing the *DAM1-4* and silencing the adjacent *DAM5-6* within the mutant *evg* locus^24^, thus providing compelling evidence that loss of expression of six *DAM* genes leads to the *EVG* phenotype. Gene expression analyses showed that in peach and other *Prunus* species, *DAM1*, *DAM2*, and *DAM4* are upregulated in apical leaf meristem during late summer and early fall, coincident with its growth cessation and bud formation, a stage of ecodormancy, while *DAM5* and *DAM6* are increased throughout fall, coincident with the transition from ecodormancy to endodormancy^25^. These data suggest that these *DAM*s differentially regulate the dormancy onset, which is further supported by a transgenic study where ectopic expression of *DAM6* in poplar promoted growth cessation, bud set and a prolonged dormancy period^26^. In almond (*Prunus dulcis*), *PdDAM6* showed a continuous decrease in transcript levels for both cultivars with different chilling requirements and flowering time during its dormancy release^27^.

Several lines of evidence also support that *DAM*s serve as direct targets of chilling temperatures. First, a major QTL trait responsible for chilling requirement was mapped to the peach *EVG* locus^28^. Second, *DAM5* and *DAM6* are downregulated during the chilling period or dormancy release^11^. Third, application of hydrogen cyanimide that promotes dormancy break in peach also downregulates *DAM5* and *DAM6* in dormant vegetative and floral buds^29^. Fourth, cultivars with a transposon insertion in both *DAM5* and *DAM6* require less chilling^30^. Thus, downregulation or genetic mutation of specific *DAM* genes is correlated to dormancy release or reduced chilling requirement. Given that *DAM*s are homologous to *Arabidopsis short vegetative phase*, a gene that codes for a transcription repressor that specifically targets, in parallel *to flowering locus C* (*FLC*), the flower and organ identify genes^31–33^, seasonal oscillation of *DAM*s could directly orchestrate the flower developmental course coupled with dormancy entry and release: Increased expression in late summer to fall slows down or arrests the floral developmental course (dormancy entry) but decreased expression during winter releases such arrest (dormancy release).

Considering variation of chilling requirement between vegetative and floral buds and male and female organs, how the cluster of six *DAM genes* in peach regulates bud- and organ-specific chilling requirement and dormancy onset and release remains unknown. In addition, whether all *DAM*s are similarly or differentially regulated in the same bud or tissues is not known. In *Arabidopsis*, chilling is known to directly target *FLC*, a transcriptional repressor, through induction of histone methylation, H3K27me3^34^, via *cold assisted intronic noncoding RNA* (*COLDAIR*) that assists the deposition of H3K27me3 on the *FLC* chromatin^35^. Whether *DAM*s share similar or different epigenetic regulation with *FLC* remains to be studied. In this study, we set out to address these questions and understand how *DAMs* are genetically and epigenetically regulated by chilling and the ensuing warm temperature regimes.

## Results

### Assessment of chilling requirement in the *evergrowing* (*EVG*) mutant peach

**Fig. 1:**
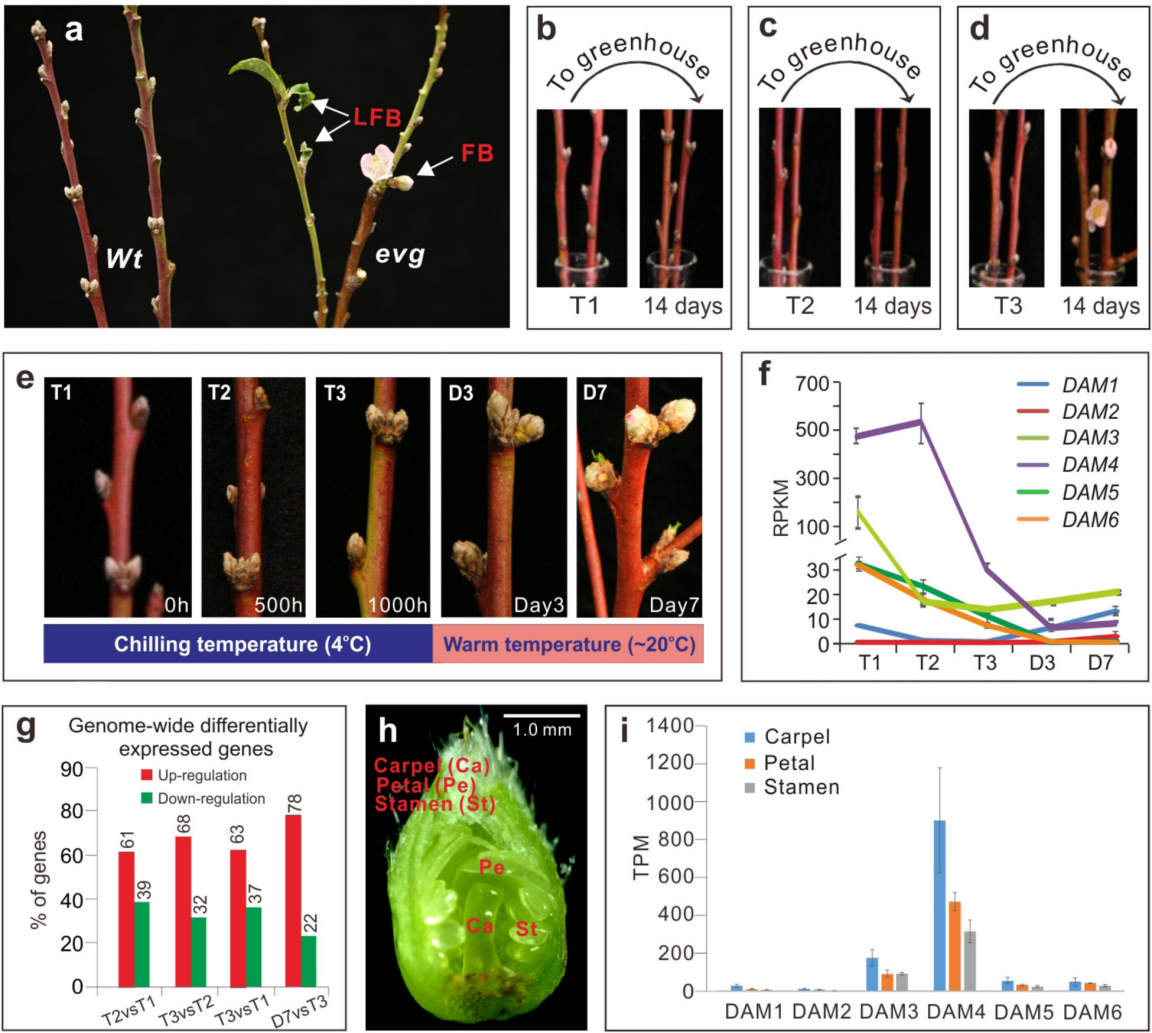
Differential regulation of six *DAM*s by chilling in dormant peach floral buds. **a** Confirmation of chilling-independent bud break in the peach *evg* mutant. The shoots attached with leaf and floral buds collected from the *evg* (right) and *Wt (left)* peach trees in the late fall were directly placed under normal growth condition (~ 20 °C) for 40 days without chilling treatment. LFB lateral leaf bud, FB floral buds. **b**–**d** In vitro chilling treatment of fully dormant *Wt* peach floral buds at 4 °C for 0 h (T1, **b**), 500 h (T2, **c**), and 1000 h (T3, **d**), and then transferred to the normal growth condition at greenhouse (~20 °C) for 14 days for assay of bud break. **e** The morphology of the chilled T1, T2, and T3 floral buds as well as the chilled T3 buds in greenhouse (~20 °C) for 3 days (D3) and 7 days (D7). **f** RNA-seq analysis of *DAM* expression plotted with RPKM (reads per kilobase per million mapped reads). Data are averaged from three biological replicates, with ±SD. **g** The percentage of total up- and downregulated genes from stage to stage. **h** Dissection and collection of petal (Pe), stamen (St), and carpel (Ca) tissues from dormant T1–T3 floral buds. **i** RNA-seq analysis of floral organ-specific expression. Data are averaged from three biological replicates, with ±SD. TPM transcripts per kilobase million mapped reads

Although the peach *EVG* mutant loses dormancy onset in the apical shoot meristem and floral buds, its lateral leaf buds appear to retain dormancy and the chilling requirement^23^, raising a question of whether *DAM*s are exclusively involved in regulation of dormancy and chilling requirement in all buds. To address this, we assessed the chilling requirement and dormancy release of the lateral leaf and floral buds by directly placing the cut shoots from the *evg* trees under permissive growth conditions (~20 °C) without chilling treatment. In parallel, the cut shoots with fully dormant leaf buds and floral buds from *Wt* peach cultivar “John Boy” were included as a control. It is noted that the *evg* tree only formed the floral and lateral leaf buds but apical meristems still remained actively growing at the time when samples were collected. Therefore, this experiment was performed on wild-type and mutant shoots with or without the removal of the apical meristems. The floral and lateral leaf buds from the *evg* tree should, if completely free from dormancy constraints, continue the course of growth or development and progress into bud break instead of arrest. Figure 1a shows that all *Wt* buds (e.g., apical and lateral leaf and floral buds) remained arrested even after 40 days under the permissive condition (left panel) unless given prior treatment of 1000 h chilling (Fig. 1b–d). But both lateral leaf and floral buds from the *evg* tree continued to break, grow, and develop (right panel, Fig. 1a). Our work provides compelling evidence that the loss of *DAM*s or their expression in the *evg* trees abolishes dormancy and chilling requirement not only in the apical shoot meristems but also in the lateral leaf and floral buds as well.

### Chilling differentially downregulated five of six *DAM*s in dormant floral buds with identification of *DAM4* as the most abundantly expressed one

To understand how the six *DAM*s were regulated by chilling during dormancy release in floral buds, we analyzed their expression and regulation in wild-type cultivar “John Boy” dormant floral buds after treatment with chilling conditions (4 °C) for 0, 500, and 1000 h (T1, T2, and T3, respectively) before being transferred to a permissive growth condition (20 °C) to induce bud break (as shown in Fig. 1b–d). This in vitro assay of the chilling requirement has been developed and used for several decades^36^, enabling accurate assessment of the effect of chilling and the following warm temperatures on dormancy release or bud break without the complications of drought, freezing, and/or transient warm spells that often occur under the field conditions. Only fully chilled T3 buds (1000 chilling hours (CH)) flowered after being transferred to the permissive growth conditions of (20 °C) in the greenhouse (Fig. 1b–d). Morphologically, the chilled T3 buds underwent slight enlargement at 3 days (D3) post warm temperature treatment and developed full petals at 7 days (D7) (Fig. 1e), indicating that 1000 CH completely releases the floral buds from dormancy constraints. On the contrary, neither T1 nor T2 chilled buds showed apparent morphological changes and did not exhibit bud break (Fig. 1b, c, e). Accordingly, we collected floral tissue (absent bud scales) from the treated buds at the T1, T2, T3, D3, and D7 stages (Fig. 1e) for RNA-seq, strand-specific RNA-seq (ssRNA-seq), small RNA-seq (sRNA-seq), whole genome bisulfite sequencing (BS-seq), and ChIP-seq analyses described below.

First, we found that chilling significantly downregulated five (*DAM1*, *3*, *4*, *5*, and *6*) of the six *DAM*s (FDR < 0.05), while *DAM2* expression was consistently low and near the limit of detection (Fig.1f). Interestingly, the five *DAM*s showed chilling stage-specific downregulation patterns. *DAM5 and DAM6* progressively decreased from T1 to T3, while *DAM1* and *DAM3* sharply dropped from T1 to T2 and *DAM4* precipitously declined from T2 to T3. The relative expression levels also varied greatly among *DAM*s. *DAM4* was the highest of all *DAM*s at T2 (~512 RPKM) followed by *DAM3* at T1 (~170 RPKM), and *DAM5* and *DAM6* at T1 (~30 RPKM), while the lowest level was reached by *DAM1* at T1 (only ~7 RPKM). Thus, the expression of *DAM4* was at least 3 times more abundant than *DAM3*, 17 times more abundant than *DAM5* and *DAM6*, and 70 times more abundant than *DAM1*. Similarly, downregulation of *DAM4* from 512 RPKM at T2 to about 30 RPKM at T3, represented about a 17-fold reduction, the most profound observed change compared to the relatively small amplitude of reduction of *DAM1*, *3*, *5*, and *6* transcript abundance. Following warm conditions (20 °C), *DAM4*, *5*, and *6* were further downregulated, while *DAM1 and 3* remained unchanged or slightly upregulated, suggesting that the warm treatment strongly reinforced the chilling-imposed repression on *DAM4*, *5*, and *6*. To rule out the possibility that repression of *DAM*s may result from ubiquitous, genome-wide transcriptional repression imposed by chilling stress, we analyzed genome-wide differentially expressed genes (DEGs) during the chilling period (Table S1). Over 60% of DEGs were upregulated by chilling from state to state (T2 vs T1, T3 vs T2, T3 vs T1, and D7 vs T3) (Fig. 1g), confirming that the downregulation of *DAM*s were biologically specific rather than result from global repression induced by chilling stress.

### *DAM3* and *DAM4* were preferentially expressed in carpel

To understand whether the *DAM* expression was potentially flower organ specific, we isolated carpel, petal, and stamen from T1 to T3 flowers (Fig. 1h), respectively, and pooled them for transcriptome analysis. Consistent with whole flower data presented in Fig. 1i, *DAM4* was the most abundantly expressed in three floral organs followed by *DAM3*, while *DAM1*, *DAM5*, and *DAM6* exhibited the lowest expression (Fig. 1i). However, expression levels of both *DAM3* and *DAM4* were almost two or three times higher in carpel tissues than petal and stamen, respectively, (Fig. 1i). Interestingly, *DAM1*, *5*, and *6*, while expressed at lower levels, had relatively higher expression in carpels.

### Differential response of sRNAs produced in the *DAM* regions to chilling and warm treatments

**Fig. 2:**
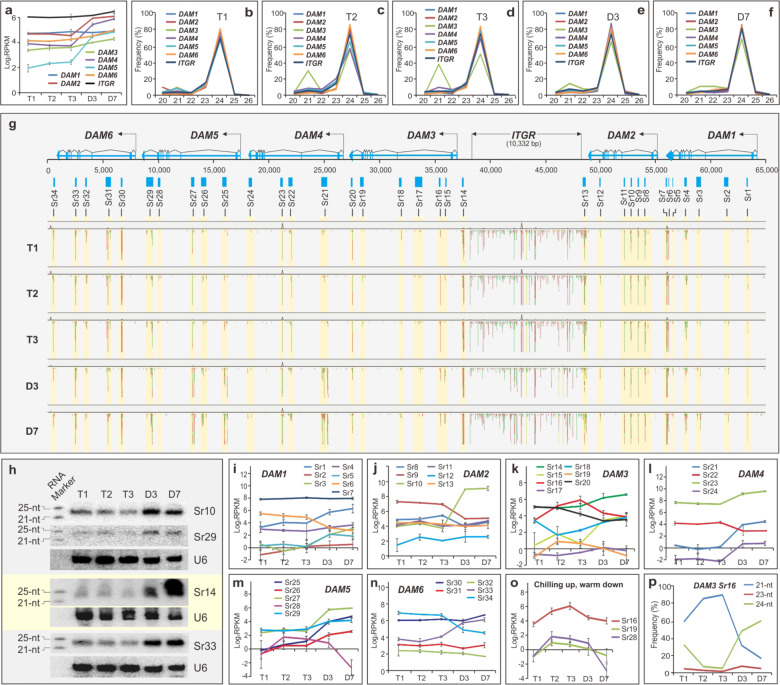
sRNA production and regulation. **a** sRNA expression in six *DAM*s. Data are averaged from three biological replicates, with ±SD. **b**–**f** sRNA population (20–26 nt) and dynamic change in *DAM*s and the intergenic region (*ITGR*) at T1 (**b**), T2 (**c**), T3 (**d**), D3 (**e**), and D7 (**f**). **g** sRNA mapping and sRNA-producing regions (*Sr*s)/loci in six *DAM* regions. The region with similar sRNA expression activity was grouped as a sRNA-producing region (*Sr*)/locus, and the 34*Sr* loci are highlighted, depicted, and marked. Red specifies antisense transcript reads and green specifies sense transcript reads. Annotated six *DAM*s and *ITGR* are depicted above. **h** RNA gel blotting. About 20 µg of enriched small RNA sample along with RNA marker was resolved on 16% of PAGE gel, blotted, probed, and reprobed with indicated p32-labeled oligos together with the labeled RNA marker. Sizes (21 and 25 nt) of sRNA markers are indicated at left and U6 serves as a control. **i**–**p** sRNA expression of individual *Sr* locus in *DAM1* (**i**), *DAM2* (**j**), *DAM3* (**k**), *DAM4* (**l**), *DAM5* (**m**), and *DAM6* (**n**), respectively. Data are averaged from three biological replicates, with ±SD. **o***Sr* loci upregulated by chilling. **p** 21-nt sRNA predominantly expressed at the *Sr16* locus

Since sRNAs are known to act as either transcriptional or post-transcriptional regulators, we next examined whether the chilling and warm temperatures also regulated sRNA production in *DAM*s. Figure 2a shows that sRNAs were produced from all six *DAM*s as well as the 10-kb intergenic region (*ITGR*) that separates *DAM1* and *2* from *DAM3*, *4*, *5*, and *6* (Fig. S1). sRNAs remained little changed from T1 to T3 in the six *DAM*s and *ITGR* region but were substantially increased in all except *DAM1* and *ITGR* from T3 to D7, indicating a warm-responsive induction. We then analyzed size (20–26 nt) of sRNAs produced in each *DAM* and their responses to the treatments. Figure 2b–f shows that 24-nt sRNA was the predominant species in all regions analyzed. Interestingly, the *DAM3* region also produced an additional 21-nt sRNA species as a minor group induced from T1 to T2 (Fig. 2b, c) and further elevated at T3 (Fig. 2d) before sharply declining at D3 (Fig. 2e), which is opposite to chilling-induced downregulation of the cognate *DAM3* (Fig. 1g). To locate where individual sRNAs were produced in *DAM*s, we mapped sRNA reads against the 65-kb *DAM* genomic region. sRNA production preferentially occurred in 34 putative *sRNA-producing regions* or loci (highlighted) that were classified based on the shared expression pattern (Fig. 2g). Seven of them were in *DAM1* and *DAM3*, six in *DAM2*, five in *DAM5* and *DAM6*, and four in *DAM4*, respectively. The *Sr* loci varied in size, ranging from 43 to 592-bp but most of them were shorter than 250-bp (Table S2). Intriguingly, only three *Sr* loci were located within the putative promoter (*Sr14* in *DAM3*) or transcriptional terminus (*Sr13* in *DAM2* and *Sr34* in *DAM6*). The remaining 31 resided within the transcribed regions. Of those 31 loci, 25 were located within introns and the remaining six resided either in the intron–exon junctions (*Sr19* in intron 7–exon 8 and *Sr20* in intron 8–exon 9 of *DAM3*, and *Sr24* in intron 8–exon 9 of *DAM4*), or in the last two exons (*Sr5*, *Sr6*, and *Sr7* in exon 9 of *DAM1*, and *Sr28* in the exon 8 of *DAM5*). To verify that *Sr* loci were independently transcribed, we performed RNA gel blotting analysis and detected 24-nt sRNA production in the representative *Sr10*, *14*, *29*, and *33* loci, respectively, but neither of them shared the same expression pattern from T1 to D7 with each other or with their cognate *DAM*s (Fig. 2h), confirming an independent regulation.

### Chilling preferentially induced 21-nt sRNA of *Sr16* located at *DAM3*, while warm upregulated many *Sr* loci coding for 24-nt sRNAs in different *DAM*s

We examined responses of the *Sr* loci to the chilling and warm treatments. First, all *Sr* loci displayed distinct expression trajectories during thermal treatment (Fig. 2i–n). Of 34*Sr* loci, 21 responded to the warm temperature from T3 to D7, with 15 upregulated (*Sr1*, *Sr3*, *Sr5*, *Sr10*, *Sr14*, *Sr15*, *Sr21*, *Sr23*, *Sr24*, *Sr25*, *Sr26*, *Sr27*, *Sr29*, *Sr30*, and *Sr33*) and six downregulated (*Sr6*, *Sr8*, *Sr9*, *Sr22*, *Sr32*, and *Sr34*), while eight (*Sr 2*, *4*, *7*, *11*, *12*, *13*, *17* and, *31*) did not respond to the chilling or warm treatments, most of which were located within *DAM1* and *DAM2*. Five *Sr* loci (*Sr16*, *18*, *19*, *20, and 28)* responded to the chilling treatment: *Sr16*, *19*, and *28* were upregulated and Sr*18* and *Sr20* downregulated. In particular, *Sr16* was the most abundant and showed the strongest response to chilling compared to others (Fig. 2o). sRNA size analysis revealed that *Sr16* was the only locus coding for a 21-nt sRNA (Fig. 2p). Hence, the chilling-induced 21-nt sRNA detected within *DAM3* from T1 to T3 (Fig. 2b–d) was exclusively encoded by *Sr16*.

### Chilling increased CHG and CHH methylation in *DAM4* but the warm treatment differentially regulated methylation at *DAM*s in a sequence context-dependent manner

**Fig. 3:**
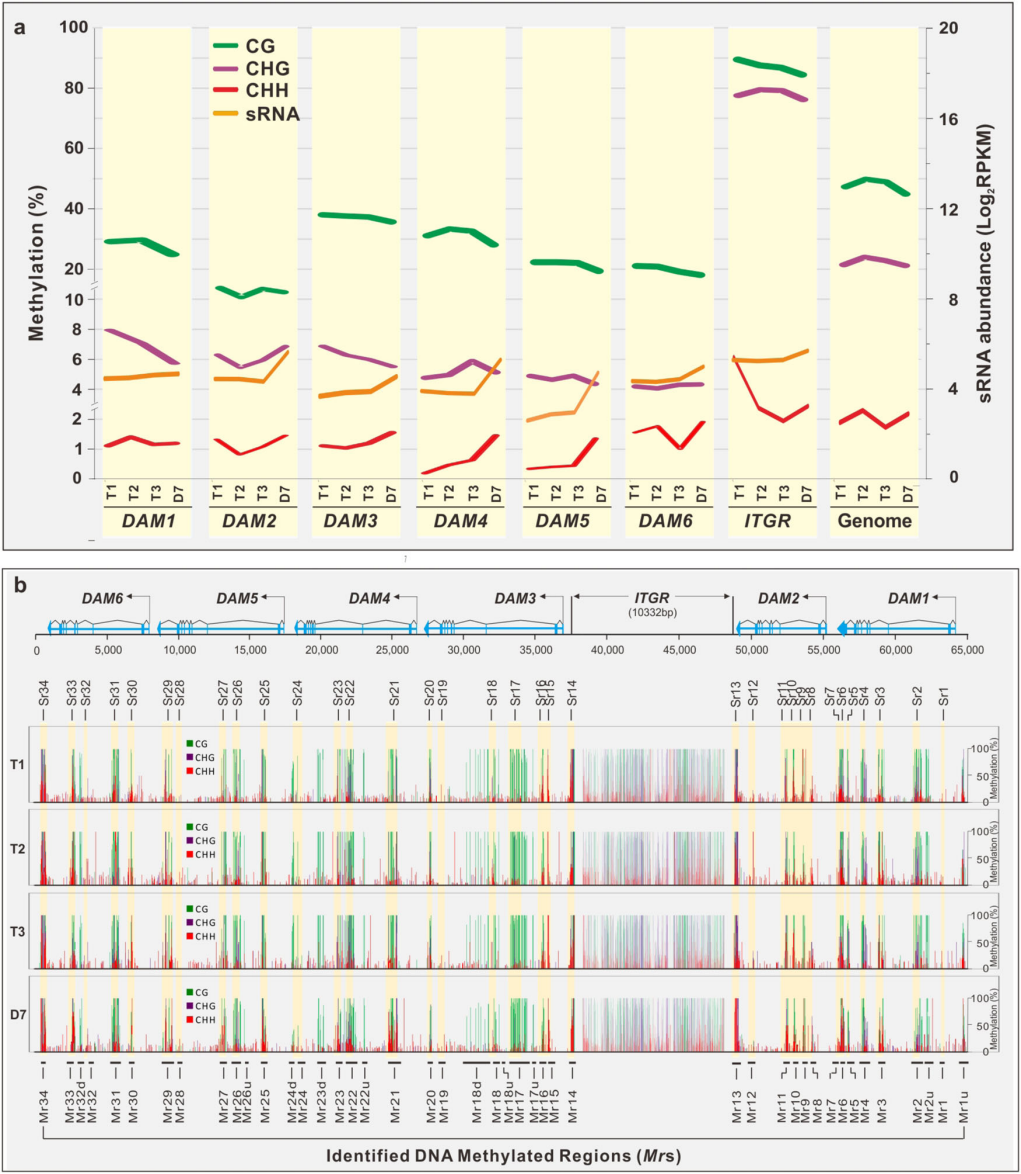
BS-seq analysis. **a** CG, CHG, and CHH methylation in six *DAM*s, *ITGR*, and peach genome. Methylation rate (%) was indicated on the left *y*-axis and sRNA abundance (orange) expressed by Log_2_ RPKM on the right *y*-axis. The treatment time point T1, T2, T3 and D7, and *DAM* genes are indicated at the bottom. **b** Cluster of methylated regions (*Mr*s). A total of 44 Methylated regions (*Mr*s) are marked in the bottom, while the corresponding *Sr* regions highlighted in yellow and denoted on top. Number of methylated regions starts from *DAM1* to *DAM6* for the sake of consistence with *DAM* position. Green—CG methylation. Purple—CHG methylation. Red—CHH methylation. The *Mr*s independent of siRNAs are named as downstream or upstream of adjacent *Mr*s (e.g. *Mr2u*, *Mr17u*, *Mr18u*, *Mr18d*, etc.)

Given that 24-nt sRNAs guide DNA methylation through RNA-dependent DNA methylation^37^, we performed whole genome BS-seq to ascertain whether chilling and warm temperatures regulate the methylation of cytosines at *DAM*s. Figure 3a shows that CG, CHG, and CHH (where H = A, T, or C) sequence contexts at *DAM*s were overall hypermethylated but the methylation trajectories responded differently to the chilling and warm temperatures. The CG methylation in all regions analyzed remained relatively constant during the chilling period (T1 to T3) but declined after shifting to the warm temperature (T3 to D7). CHH methylation changed little during chilling in all regions except *DAM4*, in which an increase in CHH methylation was observed. However, the warm temperature increased CHH methylation overall across all regions except *DAM1* and this increase was particularly pronounced in *DAM4* and *DAM5*. The increase was also correlated with an increase of sRNA production in these same *DAM*s (Fig. 3a). Evidently, the warm temperature appeared to oppositely regulate CG and CHH methylation. The effect of the chilling temperature on CHG methylation was diverse. It appeared to increase the CHG methylation in *DAM4* but decrease in *DAM1* and *DAM3*, and change little in *DAM2*, *5*, and *6*. It became apparent that *DAM4* was only gene that was up-methylated by chilling at CHH and CHG contexts and by warm at CHH context.

### Overlap of the methylated region (*Mr*) with the *Sr* region

We then investigated whether DNA methylation overlapped with the *Sr* loci or closely associated with sRNA production. Figure 3b shows that methylation was not randomly distributed in *DAM*s but rather clustered in approximately 44 regions, dubbed *Mr*s. The majority of the *Mr* loci overlapped with the *Sr* loci except *Sr1*, *19*, and *28* where there was no methylation detected. These results are indicative of widespread occurrence of RdDM in *DAM*s. The *Mr* regions were typically larger than their corresponding *Sr* loci (Table S2), consistent with methylation spreading to flanking regions^38^. Ten additional *Mr* regions (e.g., *Mr1u, Mr2u, Mr17u, Mr18u, Mr18d, Mr22u, Mr23d, Mr24d, Mr26u*, and *Mr32d*) located either upstream or downstream of the *Sr*-overlapped *Mr* loci, shared no overlap with any *Sr* region, signifying the occurrence of siRNA-independent methylation, a phenomenon frequently observed in plant genomes. Taken together, methylation regulation at the *Mr* regions in different sequence contexts under the chilling and warm conditions was diverse and complex but the overall correlation between the CHH methylation level and sRNA abundance under the warm condition was apparent in many *Mr*s (Fig. S2).

### Chilling and warm differentially induced H3K27me3 and its spreading

**Fig. 4:**
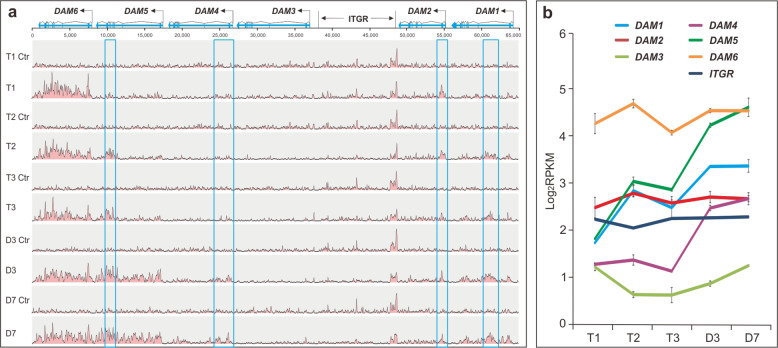
H3K27me3 in *DAM*s. **a** Genome browser tracks showing H3K27me3 ChIP-seq reads are mapped to the 65-kb *DAM* region. Five ChIP-seq tracks from T1 to D7, and their no-antibody control tracks (Ctr) are shown. The peaks in *DAM*s are marked by blue boxes. **b** Quantitative analysis of the ChIP-seq read counts in *DAM*s and *ITGR*. Data are averaged from two biological replicates, with ±SD

Earlier studies showed that chilling-induced H3K27me3 in some *DAM*s during dormancy release^39^. We performed ChIP-seq to understand how chilling and warm temperatures regulated H3K27me3 in all six *DAM*s. The ChIP-seq reads were enriched during the chilling period in *DAM1*, *2*, *5*, and *6* and some parts of the *ITGR* region but the enriched patterns and locations differed among them (Fig. 4a). Small, localized enrichment occurred in either intron or exon regions of *DAM1, 2, 5*, and a few regions of ITGR but were gene-wide in the entire *DAM6* transcribed region. Furthermore, the timeframe of the occurrence of the read enrichment varied as the enriched peak appeared only at T2 onward in *DAM1* and 5 but at T1 onward for *DAM2*, *6* and *ITGR*, indicating that the read enrichment in *DAM1* and *5* is chilling-dependent (Fig. 4a). At the warm temperature from T3 to D7, the read enrichment patterns in *DAM2* and *DAM6* along with *ITGR* remained little changed but those within *DAM1* and *5* underwent evident changes, with localized peaks becoming large to cover the entire transcribed regions, and was particularly robust in *DAM5* (bottom panels, Fig. 4a). Interestingly, *DAM4*, despite a lack of visible enrichment peak during the chilling period, also showed a major peak near the transcription start region at D3 and spread toward the 3′ end by D7 (Fig. 4a), indicating warm induced and facilitated the spreading of H3K27me across the *DAM4* region. Quantitative read enrichment analysis further confirmed that the chilling and warm conditions significantly induced and enhanced H3K27me3 in *DAM1* and *DAM5*, while warm did in the *DAM4* region (Fig. 4b). The warm temperature also enhanced H3K27me3 at *DAM3* and *DAM6* even though it was not clearly as discernable in the read map (Fig. 4a).

### Chilling induced a noncoding RNA (*ncRNA*s) in the *DAM4* region

**Fig. 5:**
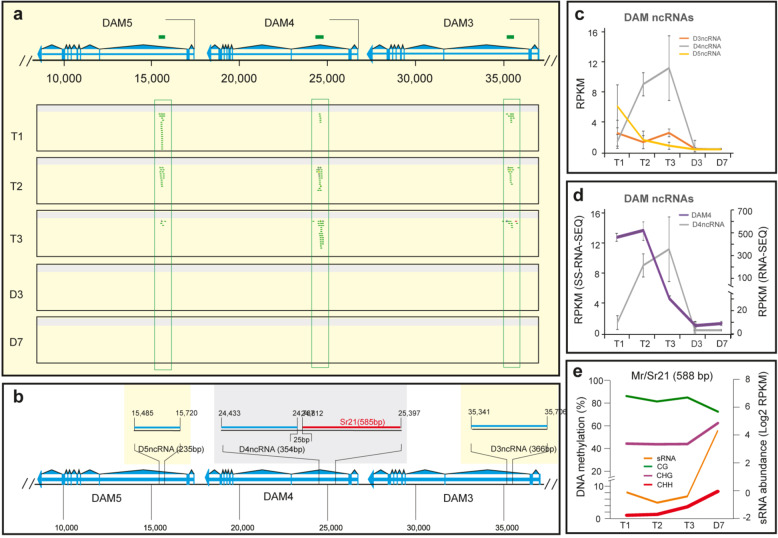
Identification of noncoding RNAs (ncRNAs). **a** Mapping of strand-specific RNA-seq reads against six *DAM*s. The sense read relevant to the *DAM* transcription direction is denoted in green and antisense in red. The detailed information about *DAM* organization and location is illustrated in Fig. S1 legend. Note that for the sake of clarity, the reads mapped to exons were removed. **b** Schematic diagram of the position and size of ncRNAs and relevant *Mr*/*Sr* locus. **c** Expression of three ncRNAs. RPKM—reads per kb per million mapped reads. **d** plot *D4ncRNA* expression from strand-specific RNA-seq data (SS-RNA-seq) with *DAM4* expression from regular RNA-seq data. **e** DNA methylation and sRNA expression in *Mr*/*Sr21* locus

Strand-specific RNA-seq was carried out to search for *ncRNA*s corresponding to *DAM*s. As shown in Fig. 5a, abundant sense reads specifically mapped to the second introns of *DAM3-5*, which roughly corresponded to 366-, 354-, and 235-nt regions, respectively, (Fig. 5b). Accordingly, we named these as putative *D3ncRNA*, *D4ncRNA*, and *D5ncRNA*, respectively. The three ncRNAs were situated at similar locations in the second intron (Fig. 5a, b) and shared ~70% of sequence identity but were differentially regulated by chilling (Fig. 5c). *D3ncRNA* and D5ncRNA remained either unchanged or downregulated, while *D4ncRNA* was drastically upregulated from T1 to T2 and reached maximal expression level at T3 (Fig. 5c). We then compared the expression of *D4ncRNA* with its cognate *DAM4* and revealed a strong inverse correlation during the chilling treatment. At the warm temperature, expression of both *DAM4* and *D4ncRNA* quickly dropped, suggesting that a common mechanism may operate to repress both at these stages. Given that chilling-inducible *COLDAIR located in the largest intron of Arabidopsis FLC* is transcribed by its own 109-bp promoter^35^, we examined epigenetic changes in the putative promoter region located upstream of the *D4ncRNA* region. Coincidently, a 588-bp *Mr21*/*Sr21* locus (described above) was located 25 bp upstream of the *D4ncRNA*-coding region and would be anticipated to overlap with the *D4ncRNA* promoter region (Fig. 5b). The *Mr21*/*Sr21* locus region also overlapped with a major peak of H3K27me3 in *DAM4* at the warm temperature (Fig. 4a). Accordingly, we focused on *Mr21*/*Sr21* and found that 24-nt sRNA expression, CHH and CHG methylation and H3K27me3 all remained at a low or a moderately low level during the chilling but were rapidly upregulated from T3 to D7 (Figs. 4a, b and 5e). Apparently, the increased sRNA, DNA methylation and H3K27me3 in the putative promoter or *Mr21*/*Sr21*, collectively correlated with a strong repression of *D4ncRNA* at the warm temperature.

## Discussion

The coupling of flower development with dormancy onset and exit cycles in response to seasonal temperatures represents a key adaptive strategy that plants evolved to cope with temperature stress in order to ensure successful reproduction. Here, we show that the chilling and successive warm temperatures regulate diverse epigenetic events that may synergically enable *DAM*s rapidly downregulated under the chilling condition and irreversibly repressed during growth seasons, allowing the flower developmental pace successfully proceeded and flower buds breaking at a proper time or season.

### Identification of *DAM4* as a key regulator in the floral buds

Earlier studies showed upregulation of *DAM1*, *2*, and *4* during the growth cessation of the shoot apical meristem and bud initiation, a stage of ecodormancy, and upregulation of *DAM5* and *6* during transition from ecodormancy to endodormancy during fall, and downregulation of *DAM5* and *DAM6* during winter period^25^. Collectively these findings suggest that these *DAM*s differentially regulate dormancy onset, development and release in apical leaf buds. In the floral and lateral buds, *DAM5* and *DAM6* were also shown to be downregulated by chilling during dormancy release^11,26,29^, suggesting that *DAM5* and *DAM6* are involved in chilling requirement and dormancy release in those buds as well. Here, our analyses revealed complex regulation of *DAM*s in peach floral buds and showed that five of the six *DAM*s were downregulated by chilling with distinct patterns (Fig. 1f). Further, we also identified *DAM4* rather than *DAM5* or *DAM6* as the most abundantly expressed *DAM* gene, with at least 3–17 times higher expression than the other four *DAM*s (Fig. 1f). *DAM4* was expressed preferentially in carpel (Fig. 1i) and was mainly downregulated at the late stage of the chilling period (Fig. 1f), which is estimated to slightly precede the formation of the ovule in the carpel, a key stage of female organ development^14–16,40^. Given the overall correlation of *DAM4* expression with dormancy exit and the corresponding unique and pronounced epigenetic events documented within *DAM4*, we propose *DAM4* as a potential key dormancy regulator in floral buds. *DAM4* exhibited 17-fold reduction of expression by chilling and remained at a considerable level (~30 RPKM) equivalent to the highest expression exhibited (~30 RPKM) by *DAM5* or *DAM6* at the T1 stage (Fig. 1f). At the warm temperature, *DAM4* continuously declined and reached the lowest level at D3 onward, indicating that the warm further downregulates *DAM4*. A predominant role of *DAM4* in floral bud dormancy and bud break is also supported by recent QTL mapping analysis^28^, which showed that Pchgms40, a marker located in *DAM4*, is more tightly linked to the traits of chilling and heat requirement and bud break compared with Pchgms12 located at D*AM6* or Pchgms41 located at *DAM5*, respectively. Taken together, *DAM4* apparently acts as a key regulator and source of trait variability for the chilling and heat requirement and bud break in peach floral buds.

It is noted that *DAM3* also shows carpel-preferential expression and to a lesser degree *DAM1*, *5*, and *6*, indicating that these *DAM*s likely play an important though lesser role in modulating dormancy and bud break phenotypes as well. The shared carpel-preferential expression among five *DAM*s would suggest that they may be similarly regulated due to their conserved sequence and duplicated nature. Given a short intergenic region (~500–720 bp) exists between adjacent *DAM*s (Fig. S1), the key regulatory elements or enhancers should, like many of them in *MADS BOX* genes^41,42^, be situated in one of the introns. Alternatively, six *DAM*s could be co-regulated by a single enhancer that activates or represses *DAM*s at a distance. This enhancer could, if potentially, reside in the *DAM4* region because deletion of *DAM1-4* in the *EVG* mutant abolishes the *DAM5* and *DAM6* expression^24^. As might be expected if this were the case, the *DAM*s flanking *DAM4* have reduced expression proportional to their distance to *DAM4* (Figs. 1f, 2g, and S1).

### Chilling drives distinct epigenetic interactions that define unique regulatory trajectories for each *DAM*

In *Arabidopsis*, chilling induces a ncRNA and H3K27me3 to silence *FLC*^34,35^. Another recent study on almond provides information on coding regions linked to early and late flowering methylation markers. It is also found that the methylation state of ten gene-coding sequences is linked to the dormancy release process^43^. In peach, chilling induces similar epigenetic responses, which vary among the six *DAM*s, with H3K27me3 induced in *DAM1*, *5*, and *6*, and *Sr16* sRNA in *DAM3* and *D4ncRNA* and CHG and CHH methylation in *DAM4*, respectively. None of these *DAM*s shares the same epigenetic regulation with each other or with *Arabidopsis FLC*, indicating *DAM*-specific epigenetic regulation. However, all *DAM*s are ubiquitously hypermethylated (Fig. 3a, b), and such hypermethylation may add an additional layer of repression for each *DAM*. Hence, interactions between hypermethylation and other epigenetic elements should contribute to variation of expression abundance and pattern among *DAM*s.

Previous work showed that H3K27me3 in *DAM1, 4, 5*, and *6* in floral buds is upregulated during dormancy release under field conditions^39^, but the absence of clearly defined chilling and warm periods and dormancy state makes it hard to discriminate the role of chilling from the successive warm temperature. Our work shows that chilling and warming function distinctly: Chilling induces the localized H3K27me3 in *DAM1* and *5*, while the warm enhances and spreads it gene-wide. In *DAM4*, only warm but not chilling induces H3K27me3. However, H3K27me3 in *DAM6* occurs before chilling treatment and remains almost unchanged from the beginning of chilling to end of the warm period (Fig. 4a, b), apparently contradicting with the earlier finding that H3K27me3 in *DAM6* is upregulated during dormancy release^39,44^. Hence, the chilling and warming effect on H3H27me3 varies among *DAM*s and possibly differently among peach cultivars. Interestingly, the presence of H3K27me3 is, regardless of abundance and stages, associated with lower expression (e.g. *DAM1*, *5*, and *6*), while the absence of it is correlated with higher expression (e.g. *DAM3* and *4*) during chilling period (Fig. 1f). This could be interpreted to imply that H3K27me3 presents an overall repressive effect on *DAM*s or that the lower expressed *DAM*s may be readily prone to H3K27me3.

Chilling-induced *COLDAIR* in the *Arabidopsis FLC* is involved in recruitment of a protein complex that deposits H3K27me3^35^, but lack of detectable H3K27me3 in *DAM4* during the chilling period indicates that the chilling-induced *D4ncRNA* functions differently in *DAM4*. ncRNAs have been shown to regulate H3K9 methylation^45,46^, histone deacylation,^47^ and recruitment of protein or transcription factors^48,49^. *D4ncRNA* may be involved in one of these regulatory events to downregulate *DAM4*. The role of the chilling-induced 21-nt sRNA coded by *Sr16* in *DAM3* remains mysterious and it may directly or indirectly repress the *DAM3* expression transcriptionally or post-transcriptionally.

### Warm treatment reinforces the chilling-imposed epigenetic repression on *DAM*s

The fact that warming reinforces the chilling-induced, localized H3K27me3 in *DAM*s (Fig. 4a, b) is consistent with the effect of warming on enhancement of the chilling-induced H3K27me3 in *FLC* in the vernalized *Arabidopsis* plants^50,51^. Such enhancement is believed to stabilize and reinforce repression of *FLC* over the growing season^52^. Expectedly, the warm-induced upregulation of H3K27me3 in peach floral buds also reinforces the repression of the *DAM* genes. In addition, we also show that warming corresponds with upregulation of the CHH methylation, which is particularly apparent in *DAM4*, *5*, and *6* (Fig. 3a–d). The increased methylation is correlated with 24-nt sRNA expression, indicating that warming may activate RdDM in *DAM*s. The concurrence of H3K27me3 with CHH methylation in the same *DAM*s should synergistically impose a stronger repression than either alone, which is supported by continuous downregulation of *DAM4* and steadily repression of *DAM5* and 6 during the warm period (Fig. 1f) because all three *DAM*s are subjected to stronger H3K27me3 and CHH methylation compared with *DAM1*, *2* and *3* (Figs. 3a and 4a, b).

### The warm-induced and reinforced epigenetic repression on *DAM*s is biologically important

In the winter *Arabidopsis* ecotype, a few weeks of a warm period (~20 °C) immediately following vernalization/chilling treatment is critical for establishing and stabilizing vernalization/chilling effects^50^. The vernalized/chilled plants lose their commitment to flowering when immediately placed at 30 °C but remains committed to flowering when placed at 20 °C for 2 weeks before being transferred to 30 °C. Thus, the warm period at 20 °C following vernalization/chilling is critical for stabilizing or reinforcing “the vernalized state.” A similar phenomenon is also observed in peach flower buds^21^. The fully chilled flower buds usually develop normally at the warm temperature at or below 20 °C but abnormally at or above 25 °C. The higher temperature often causes the arrest of reproductive organs especially the embryo sac and results in poor fruit set^21^. Molecular analysis revealed that the chilling-induced H3K27me3 at the *FLC* chromatin in the vernalized plants is strongly enhanced at the ensuing warm treatment (20 °C or below) but substantially reduced at 30 °C unless prior exposure to 20 °C for 2 weeks^50,51^, which supports the role of warming (20 °C) in epigenetic reinforcement of the chilling-induced vernalization state. Hence, the warm-enhanced H3K27me3 and CHH methylation in the peach floral buds should similarly impose a stronger and stable repression on *DAM*s, but higher temperatures (≥25 °C) could abrogate such repression, leading to ectopic expression of *DAM*s in carpels and compromising the formation or development of normal embryo sac and other tissues^21^. To this end, the rate and efficiency of epigenetic response to the warm temperature to achieve a strong and irreversible repression on *DAM*s could vary among different species or cultivars, resulting in the different warm period or total heat sum required for bud break or flowering.

## Materials and methods

### Chilling treatment of dormant peach floral buds

Shoots from peach cultivar “John Boy” (*Wt*) and *EVG* were collected from trees grown in USDA-ARS orchard located at Kearneysville, WV, at the end of October when full dormant state in the buds are developed. The collected shoots were directly placed at 20 °C for assay of bud break for up to 8 weeks, or in containers filled with 1/5 water and chilled at 4 °C in the growth chamber in dark for 0, 500, and 1000 h (CH). The flower tissues were collected by dissecting and removing bud scales that enclose the buds, at three different stages: 0 (T1), 500 CH (T2), and 1000 CH (T3). After chilling treatment, the shoots were placed in the greenhouse (~20 °C) for assay of bud break, and the flower buds from shoots kept for 3 (D3) and 7 (D7) days in the greenhouse were collected. Three replicates were conducted for each time point of sample collection. The same tissues were used for isolation of RNA and DNA for RNA-seq, BS-seq, sRNA-seq, and ChIP-seq analyses described below. The petal, carpel, and stamen tissues were also dissected and isolated from T1, T2, and T3 buds and pooled the same tissue together for analysis of floral organ-specific regulation of *DAM*s.

### RNA-seq and strand-specific RNA-seq data analyses

Total RNA samples with three biological replicates were isolated from the flower tissues or organ tissues. About 200 mg of the floral tissues were grounded in liquid nitrogen and extracted using TriReagent (Sigma, St Louis, MO, USA) followed by two rounds of phenol–chloroform extraction (50:50). About 5 μg of each sample was submitted to BGI Americas Cooperation (Cambridge, MA, USA) for RNA-seq and ssRNA-seq using Illumina Hi-Seq. RNA-seq reads were first processed by removing the 3′ adapter sequence, filtering out rRNA and tRNA sequences via CLC Genomic Workbench V.5, 20 (Qiagen, Hilden, Germany). Floral organ-specific expression and analysis of ncRNAs and expression were conducted by directly mapping the resulting filtered reads to the 65-kb region accurately annotated with the six *DAM*s based on Peach genome 1.0^53^ (also see Fig. S1). The read counts in each *DAM*s or *ncRNA* region were normalized to reads per transcript per million mapped reads or reads per kilobase of exon model per million mapped reads (RPKM), respectively. Differential gene and transcript expression analysis were conducted according to instruction provided by CLC Bio (Qiagen, Hilden, Germany), and Raw *P* values of multiple tests were corrected using FDR^54^.

### ChIP-seq and analysis of H3K27me3 in *DAM*s

About 200–300 mg of the harvested peach flower bud tissues were ground to fine power under liquid nitrogen, and the nuclei isolation and ChIP-seq were performed as previously described^55^. The isolated chromatins were digested by micrococcal nuclease (NEB, Ipswich, MA, USA). Half of the nucleosomes was directly used for library construction and sequenced as a negative control, while the other half was subjected to immunoprecipitation using antibody against H3K27me3 (Millipore 07-449). About 0.5 to 1 µg of the recovered immunoprecipitated DNA samples were submitted to the core facility of Cornell Weill Medical College for library construction and sequencing on HiSeq2000 platform. Two replicates for each sample were performed. The ChIP-seq reads were mapped using bowtie and peak calling was performed using MACS2 and CLC platform (Qiagen, Hilden, Germany). The reads per kilobase per million mapped reads (RPKM) were calculated and statistically analyzed as presented in Fig. 4b.

### RNA Gel blotting

RNA blot analysis was carried out as described previously by Zhu et al^56^. Briefly, total RNA was extracted from peach floral buds using TriReagent (Sigma, St Louis, MO, USA) followed by two rounds of phenol–chloroform extraction (50:50). sRNAs were further enriched using the mirVana miRNA isolation kit (AM1560, Thermo Fisher Scientific), and about 25 µg of the enriched sRNA was blotted on to the nylon membrane filters, which were probed and re-probed with ^32^P-labeled *Sr*-specific probes together with the sRNA Marker Probe labeled with γ^32^P-ATP using T4 polynucleotide kinase (NEB, Beverly, MA, USA).

### Small RNA sequencing and read alignment

Small RNAs were isolated and enriched from total RNA as described above, and ligated to a 5′ RNA adapter and a 3′ RNA adapter, as described previously^57^. The ligation product was RT-PCR amplified and gel purified before sequencing on Illumina HiSeq 2000 platform. Three biological replicates were sequenced. Adapter sequences were first removed from raw sRNA reads. The resulting sRNA sequences were further processed to remove those containing low-complexity and t/rRNA sequences, and having lengths <15 bp or >29 bp. The remaining high-quality sRNA reads were aligned to the peach genome 1.0 and the 65-kb DAM sequence with perfect matches and reads with multiple alignments in the genome were excluded from further analysis. Raw read counts for each sRNA were normalized to RPKM and statistical analysis of changes of all sRNAs along *DAM* region (*Sr*) during temperature-dependent dormancy release and flowering was performed, using CLC Genomic Workbench V.5 (Qiagen, Hilden, Germany).

### Whole genome bisulfite sequencing and data processing

DNA samples with three replicates were isolated from flower tissues using the DNeasy^®^ Plant Mini Kit (Qiagen, Hilden, Germany) and submitted to BGI Americas Cooperation (Cambridge, MA, USA) for whole genome BS-Seq, with about 30X genome coverage. The error conversion rates of the BS sequences were below 0.005% for unmodified cytosines, comparable to previous data^58^. To align the BS-seq reads to peach genome, cytosine bases in the reads were first replaced with thymines. The converted reads were then aligned to the computationally converted strands of the peach genome 1.0 (one with C to T and the other with G to A), respectively, using the Bowtie algorithm allowing up to two mismatches^59^. Alignments from both strands were combined, and for each read only the optimal alignments were kept. Multialigned reads were not included in the analysis. The read sequences in the alignments were then replaced with the original, nonconverted sequences^59^. Finally, methylation level of each cytosine was calculated genome-wide and the methylation profiling (in CG, CHG, and CHH contexts) was presented along *DAM* region, on the basis of alignments.

## Supplementary information

Supplementary Table S1

Supplementary Table S2

Supplementary Figure S1

Supplementary Figure S2

## Data Availability

All the raw data from this study have been submitted to the NCBI BioProject database (http://www.ncbi.nlm.nih.gov/bioproject/493230) under accession number PRJNA493230.
