# Supplementary figures and images for: Thermal-responsive genetic and epigenetic regulation of DAM cluster controlling dormancy and chilling requirement in peach floral buds
Source: Hortic Res. 2020 Aug 1;7:114. doi: 10.1038/s41438-020-0336-y (PMC7395172; doi:10.1038/s41438-020-0336-y)

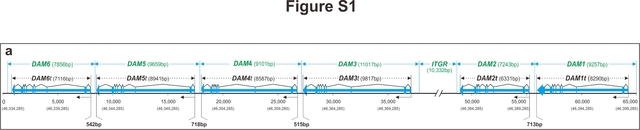

Supplement: Supplementary file 3 — Supplementary Figure S1 [file 41438_2020_336_MOESM3_ESM.jpg]

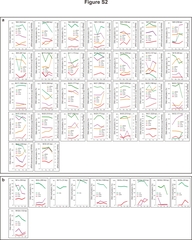

Supplement: Supplementary file 4 — Supplementary Figure S2 [file 41438_2020_336_MOESM4_ESM.jpg]
